# Supplementary material for: MetaRibo-Seq measures translation in microbiomes
Source: Nat Commun. 2020 Jun 29;11:3268. doi: 10.1038/s41467-020-17081-z (PMC7324362; doi:10.1038/s41467-020-17081-z)
Supplement: Supplementary file 10 — Supplementary Data 7 [file 41467_2020_17081_MOESM10_ESM.zip › File2/Confidence_High_Taxonomy/337350_out.krona.html]

Javascript must be enabled to view this page.

members
magnitude
magnitudeUnassigned
count
unassigned
taxon
rank

337350\_out

4

superkingdom
2
4

4
1239
phylum

186801
class
4

186802
order
4

31979
family
4

1485
genus
4

species
1262778

SRS019068\_contig\_number\_85421SRS042966\_contig\_number\_10286SRS049773\_contig\_number\_29388SRS143466\_contig\_number\_8857
4
